# Increasing abscisic acid levels by immunomodulation in barley grains induces precocious maturation without changing grain composition

**DOI:** 10.1093/jxb/erw102

**Published:** 2016-03-07

**Authors:** Nicole Staroske, Udo Conrad, Jochen Kumlehn, Götz Hensel, Ruslana Radchuk, Alexander Erban, Joachim Kopka, Winfriede Weschke, Hans Weber

**Affiliations:** ^1^Leibniz-Institut für Pflanzengenetik und Kulturpflanzenforschung, D–06466 Gatersleben, Germany; ^2^Max-Planck-Institute of Molecular Plant Physiology, D-14476 Potsdam-Golm, Germany

**Keywords:** Abscisic acid, barley, grain composition, grain maturation, immunomodulation, seed development, stress signalling.

## Abstract

Abscisic acid (ABA) accumulates in seeds during the transition to the seed filling phase. ABA triggers seed maturation, storage activity, and stress signalling and tolerance. Immunomodulation was used to alter the ABA status in barley grains, with the resulting transgenic caryopses responding to the anti-ABA antibody gene expression with increased accumulation of ABA. Calculation of free versus antibody-bound ABA reveals large excess of free ABA, increasing signficantly in caryopses from 10 days after fertilization. Metabolite and transcript profiling in anti-ABA grains expose triggered and enhanced ABA-functions such as transcriptional up-regulation of sucrose-to-starch metabolism, storage protein synthesis and ABA-related signal transduction. Thus, enhanced ABA during transition phases induces precocious maturation but negatively interferes with growth and development. Anti-ABA grains display broad constitutive gene induction related to biotic and abiotic stresses. Most of these genes are ABA- and/or stress-inducible, including alcohol and aldehyde dehydrogenases, peroxidases, chaperones, glutathione-S-transferase, drought- and salt-inducible proteins. Conclusively, ABA immunomodulation results in precocious ABA accumulation that generates an integrated response of stress and maturation. Repression of ABA signalling, occurring in anti-ABA grains, potentially antagonizes effects caused by overshooting production. Finally, mature grain weight and composition are unchanged in anti-ABA plants, although germination is somewhat delayed. This indicates that anti-ABA caryopses induce specific mechanisms to desensitize ABA signalling efficiently, which finally yields mature grains with nearly unchanged dry weight and composition. Such compensation implicates the enormous physiological and metabolic flexibilities of barley grains to adjust effects of unnaturally high ABA amounts in order to ensure and maintain proper grain development.

## Highlight

Increasing ABA by immunomodulation in barley grains induces precocious maturation, stress signalling and mechanisms to desensitize ABA signalling, which ultimately leads to unchanged grain weight and composition.

## Introduction

In barley, endosperm cellularization is completed at 5–6 days after fertilization (DAF). At 10 DAF, the endosperm begins to accumulate storage products. The pre-storage phase from anthesis until 6 DAF and the storage phase from 10 DAF onwards are separated by a transition stage characterized by transcriptional reprogramming, which promotes the switch of the endosperm into the storage mode. Grain dessication starts at physiological maturity after 20 DAF and grains are fully mature at around 40 DAF (Sreenivasulu *et al.*, 2006).

Abscisic acid (ABA) in plants regulates many developmental processes and responses to environmental stress. In seeds, it is necessary for maturation such as storage product accumulation, desiccation tolerance and dormancy (Finkelstein *et al.*, 2008; Cutler *et al.*, 2010). Levels of ABA are low during early embryogenesis but increase considerably at the onset of maturation, which is generally correlated with the physiological status of seeds. The transition of seeds from pre-storage to maturation is partially controlled by ABA and a network of sugars and ABA (Finkelstein and Gibson, 2002; Weber *et al.*, 2005; Radchuk *et al.*, 2010b). ABA functions in context with other phytohormones. For instance, ABA and gibberellic acids (GAs) are negatively correlated during seed growth and levels fluctuate during seed development, implicating a tightly regulated balance (White *et al.*, 2000; Weier *et al.*, 2014). While GAs stimulate growth by cell elongation (Thiel *et al.*, 2008), ABA functions antagonistically and generally inhibits growth and cell elongation at higher concentrations but is necessary for seed maturation events (Weber *et al.* 2005) including sugar signalling (Finkelstein and Gibson, 2002). A plethora of different genes and second messengers are involved in ABA signalling, such as phospholipases, protein kinases/phosphatases, mitogen-activated kinase, sucrose non-fermenting 1-related kinase 1 (SnRK1), phosphatidic acid, reactive oxygen species and nitric oxide (Hirayama and Shinozaki, 2007). Multiple receptors for ABA have been identified (Ma *et al.*, 2009; Fuchs *et al.*, 2014). While different receptors perceive ABA signals (Boursiac *et al.*, 2013; Nakashima and Yamaguchi-Shinozaki, 2013; Xu *et al.*, 2013), no single receptor acts upstream of the signalling pathway, suggesting that ABA responses are mediated by distinct receptor classes and that signalling components depend on cell type and physiological processes and development (Verslues and Zhu, 2007; Spartz and Gray, 2008).

Plant hormone immunomodulation can alter the hormonal status without directly intervening with its synthesis, degradation or signalling pathways and as such could represent a suitable tool to directly study the functions of ABA during seed maturation (Conrad and Manteuffel, 2001). Correspondingly, an ABA-specific single-chain antibody (scFv) was directed into the endoplasmic reticulum (ER) lumen of tobacco seeds, resulting in a developmental switch from maturation to germination, which is characterized by chloroplast formation and reduction of storage oil and protein content (Phillips *et al.*, 1997). The antibody, located in the ER, binds ABA, and as such represents an ABA sink in this compartment (Strauss *et al.*, 2001). Thereby, expression of the ABA-binding single-chain antibody impacts the subcellular distribution of ABA, generating a phenotype similar to the Arabidopsis *aba/abi3* double mutant seeds (Koornneef *et al.*, 1989) and thus delays the concentration of ABA in compartments other than the ER. Immunomodulation in pea embryos by overexpression of an anti-ABA antibody reduces free ABA levels specifically during the transition from pre-storage to storage phase (Radchuk *et al.*, 2010a). This deficiency of free ABA decreases accumulation of seed dry matter, delays the differentiation process and generally down-regulates gene expression related to transcription and translation. These anti-ABA embryos exhibit a widespread repression of sugar uptake and metabolism, diminished starch, amino acid and storage protein biosynthesis. The interaction of ABA with SnRK1 complexes highlights the cooperation between SnRK1, sugar, stress and ABA signalling (Radchuk *et al.*, 2010a).

In this work, we aimed to analyse the effect of ABA immunomodulation on grain development and maturation in barley. Similar to the situation in tobacco, Arabidopsis and pea, ABA immunomodulation drastically increases ABA in barley caryopses from the transition stage onwards. However, it was calculated that the antibody only binds a small fraction of this ABA in the transgenic caryopses, which results in strongly enhanced levels of free ABA. This renders these grains as suitable models to analyse the influence of increased ABA levels on transcriptional and metabolic control of grain development. Metabolite and transcript profiling in these anti-ABA grains expose up-regulation of signal transduction and stress response especially during the transition phase, which is consistent with triggered, enhanced and precocious initiation of maturation processes. The fact that weight and composition of ripe grains remain unchanged indicates that barley caryopses induce specific mechanisms to desensitize ABA signalling. Such mechanisms point to the enormous physiological and metabolic flexibility of barley grains to adjust extreme internal effects in order to ensure and maintain proper grain development.

## Materials and methods

### Plant material, transformation and quantitative PCR

Barley (*Hordeum vulgare* L. cv. Igri) was grown in greenhouses with 16/8h light/dark at 19/14 °C during the generative phase. Stages of grain development were determined as described previously (Weschke *et al.*, 2000). Plant tissue was collected between 10 and 12 am at 3, 7, 10, 14, 20 DAF. Filial grain fractions were manually separated from the pericarp between 7 and 14 DAP, and whole caryopses were sampled at 20 DAF.

Barley plants (*Hordeum vulgare*, cv. Igri) were transformed with an anti-ABA single-chain variable fragment (scFv) gene (Artsaenko *et al.* 1995). Plant transformation was based on infection of embryogenic pollen cultures with *Agrobacterium tumefaciens* (Kumlehn *et al.*, 2006), which can result in instantaneously homozygous plants with regard to the transgene, provided whole genome duplication occurs spontaneously or is chemically induced after transgene integration. In order to achieve grain-specific immunomodulation, the 1Ax1 promoter was used (Halford *et al.*, 1992).

Towards constructing 1Ax1:GFP plants: a *Not*I/*Xba*I 1Ax1::GFP fragment was released from WBVec8/1Ax1::GFP and introduced into the corresponding sites of pNOS-AB-M (DNA Cloning Service, Hamburg, Germany) to generate p1Ax1::GFP-Nos. Subsequently a *Sfi*I fragment harbouring the entire expression cassette was sub-cloned into p6U (DNA Cloning Service, Hamburg, Germany) to generate constructs p6U-1Ax1-GFP. The binary plasmids were introduced into *A. tumefaciens* strain LBA4404pSB1 (Komari *et al.*, 1996). In total eight independent transgenic lines were regenerated with transgene inserts. Three stable homozygous lines – 362, 363 and 364 – were chosen for further analysis.

Copy number of the aABA scFv gene was determined by genomic Southern Blot analysis (Supplementary Fig. S1, available at *JXB* online). 10 μg of genomic DNA from leaves were cut by BamHI and HindIII, separated by electrophoresis and immobilized on nylon filters. Hybridization was done under stringent conditions using a 447bp fragment of the anti-ABA scFv gene labelled with ^32^P. Copy number was verified by quantitative PCR (Thermocycler 7900HT, Applied Biosystems) using 5ng genomic DNA. First strand synthesis was done using Superscript^TM^III (Invitrogen). Gene-specific primers were: actin 1211rev 5ʹ-AGC ACT TCC GGT GGA CAA T-3ʹ , actin 1153 fwd 5ʹ-GTG GAT CTC GAA GGG TGA GT-3ʹ, aABA-681fwd 5ʹ-TGG CAG TGG GTC AGG AAC TA-3ʹ, aABA-738rev 5ʹ-ATC CTC AGC CTC CAC TCT AC-3ʹ. All reactions were performed using Power SYBR Green PCR Master Mix (Applied Biosystems).

### Germination assay

Eight replicates of each 25 grains were tested in a standard germination assay (ISTA 2008 international rules for seed testing, International Seed Testing Association, Bassersdorf, Switzerland). The grains were incubated in trays between two layers of wet filter paper and incubated in a light chamber at a day/night temperature of 20/18 °C (14h light). Regularly, the number of germinated grains was determined by counting. Grains that did not germinate after 12 d were regarded as dormant.

### Determination of anti-ABA scFv content, calculation of free ABA, and measurement of ABA

Estimation of anti-ABA scFv antibody protein content was performed by western blot analysis as described previously (Fiedler and Conrad, 1995). Determination of the dissociation constant, Kd, of the anti-ABA scFv antibody purified from anti-ABA barley grains with free ABA was done by competition ELISA of affinity-purified scFv protein (Artsaenko *et al.* 1995, Radchuk *et al.* 2010a). Levels of free ABA were calculated using the equation Kd=(scFv)(ABA)/(scFv-ABA complex), (Neri *et al.*, 1996), for different time points and by subtracting the concentration of (scFv−ABA complex) from the total ABA amount. ABA was extracted from plant material and analysed as described previously (Miersch *et al.*, 2008).

### RNA isolation, labelling, array hybridization, and data evaluation

Grain material from three biological replicates was harvested at 7, 10, 14 and 20 DAF; and total RNA was extracted from the filial fractions using the Gentra RNA Isolation Kit (Biozyme Scientific, Oldendorf, Germany). RNA integrity was confirmed using the Bioanalyser system (Agilent Technologies). 100ng RNA was used for cRNA synthesis and Cy3-labelling with a Low Input Quick Amp Labelling Kit (Agilent Technologies, www.agilent.com). Labelling efficiency, and amount and quality of cRNA were assured using an ND-1 000 Spectrophotometer (NanoDrop Technologies, Wilmington, USA) and Bioanalyser system. 600ng labelled cRNA was used for fragmentation and array loading (Gene Expression Hybridization Kit, Agilent Technologies, www.agilent.com). Hybridization of the AGILENT Barley Gene Expression Microarray, 4×44K (www.agilent.com) was performed by Atlas Biolabs GmbH, Berlin, Germany. Resulting images were evaluated (determination of spot intensities, background correction) with Feature Extraction V11.5 (Agilent Technologies, www.agilent.com). For further evaluation only spots were considered whose signal intensity was significantly different by at least 2-fold (*t*<0.05; moderated *t*-test), (Benjamini and Hochberg, 1995) between wild type and transgenic line. All sequences fulfilling these criteria were subjected to BLAST×2 searches against the NRPEO-data base (ftp://ftp.ncbi.nih.gov/blast/db/FASTA/nr.gz) using the HUSAR software (http://genome.dkfz-heidelberg.de).

### Determination of sucrose, starch, globulins/albumins, free amino acids, total carbon, and nitrogen and metabolite profiling

After tissue preparation all samples were immediately frozen in liquid nitrogen. Extraction and determination of glucose, fructose, sucrose, starch and globulins/albumins were performed as described (Rolletschek *et al.*, 2002). Free amino acids were performed as described (Thiel *et al.*, 2009). Relative contents of total carbon and nitrogen in dried, powdered samples of grains were measured using an elemental analyser (Vario EL; Elementar Analysensysteme, http://www.elementar.de/cms/en/home/). Statistical analysis was performed using Sigma Stat software (SPSS, http://www.systat.de.).

For GC-MS measurements of polar central metabolites, 30mg of fresh material of filial fractions of grains from line 363 and wild-type Igri, were harvested with six biological replicates at 7, 10, 14 and 20 DAF. Sample preparation, extraction and data evaluation were carried out as described (Erban *et al.*, 2007; Weichert *et al.*, 2010) except that a 0.007mg ml^−1^ stock solution of ^13^C6-sorbitol was used as internal standard. The internal standard concentration was reduced to one third, i.e. 500 μl, due to the high volumes of dried polar extract that were analysed in this study. Samples were profiled by GC-MS in splitless injection mode and, for the proofing of highly concentrated metabolites, in 1:30 split mode. Metabolite data were normalized to sample fresh weight and internal standard and are presented as log2-transformed pool size ratios of line 363 over wild type. Calculations, heat map formatting, and two-sample significance testing were performed using Microsoft Excel software assuming heteroscedastic variance of log2-transformed ratios. *P*-values were corrected for multiple testing (Benjamini and Hochberg, 1995).

## Results

### Plant transformation and scFv-transgene expression

**Fig. 1.**
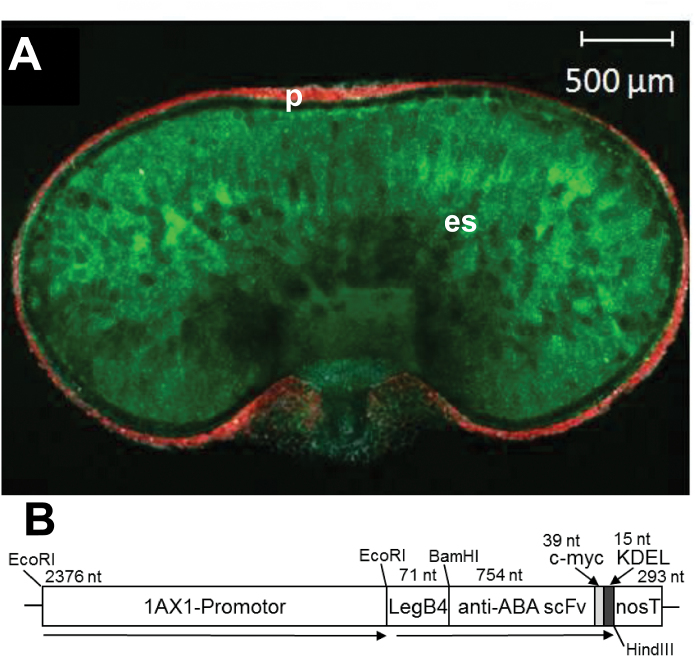
Activity of the 1Ax1-promoter. (A) Green-fluorescence-protein (GFP, green) und chlorophyll-fluorescence (red) in cross-sections of developing barley caryopses, expressing GFP under control of the 1Ax1 promoter from wheat and showing specific activity within the endosperm during the storage phase. es, endosperm; p, pericarp. (B) Schematic view of the anti-ABA scFv-construct. For grain-specific expression the 1Ax1 promoter from wheat was used. The LeguminB4-signal peptide (LeB4) and the ER-retention signal (KDEL) mediate scFv accumulation in the ER. The c-myc tag allows immuno-detection of the anti-ABA scFv.

**Fig. 2.**
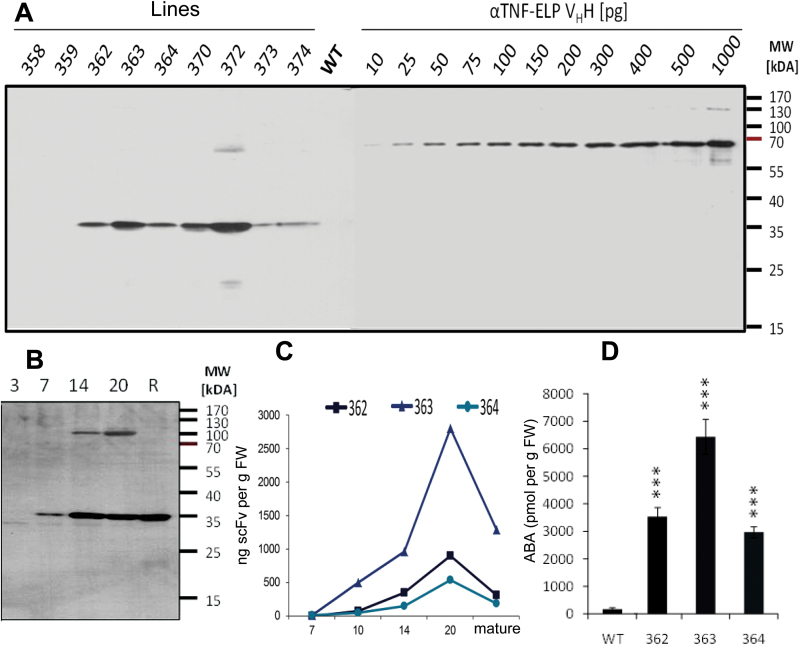
Transgene expression and ABA accumulation. (A) Determination of anti-ABA scFv-protein accumulation in mature caryopses of transformands. 30 µg raw protein and different amounts of αTNF-ELP V_H_H-antibody were loaded per lane. After separation (12% SDS-PAA-Gel, reducing conditions) detection was performed by western blot using anti-c-myc antibody followed by anti-mouse IgG peroxidase and ECL-reaction. (B) Amounts of anti-ABA scFv in developing caryopsis at 3, 7, 14 20 DAF and at maturity (R). (C) Semi-quantification of anti-ABA scFv accumulation in lines 362, 363 and 364. (D) ABA accumulation in mature caryopses. ***, significant differences compared to the wild type, *P*<0.001. (This figure is available in colour at *JXB* online.)

Transgenic barley expressed the scFv-gene under control of the 1Ax1-promoter, which is strongly active in the endosperm as shown by the expression of a 1Ax1-promoter: green-fluorescent-gene (GFP) fusion, (Fig. 1A). The scFv expression unit includes the LeB4 signal peptide, the endoplasmatic reticulum retention signal (KDEL) and the *c-myc*-tag (Munro and Pelham, 1987) (Fig. 1B). Accumulation of anti-ABA scFv-protein was determined semi-quantitatively in developing and mature caryopses of primary transformands of the anti-ABA plants and in developing caryopses using the *c-myc*-tag and standard proteins (Conrad *et al.*, 2011) (Fig. 2A). In three lines, scFv-protein accumulated from 7 DAF to highest levels at 20 DAF (Fig. 2B, C). These three stable homozygous lines – 362, 363 and 364 – were chosen for further analysis. Transgene copy number of the lines was determined by quantitative PCR and yielded two, four and two copies for lines 362, 363 and 364.

### Anti-ABA antibody expression interferes with accumulation of free unbound ABA in the grain

**Fig. 3.**
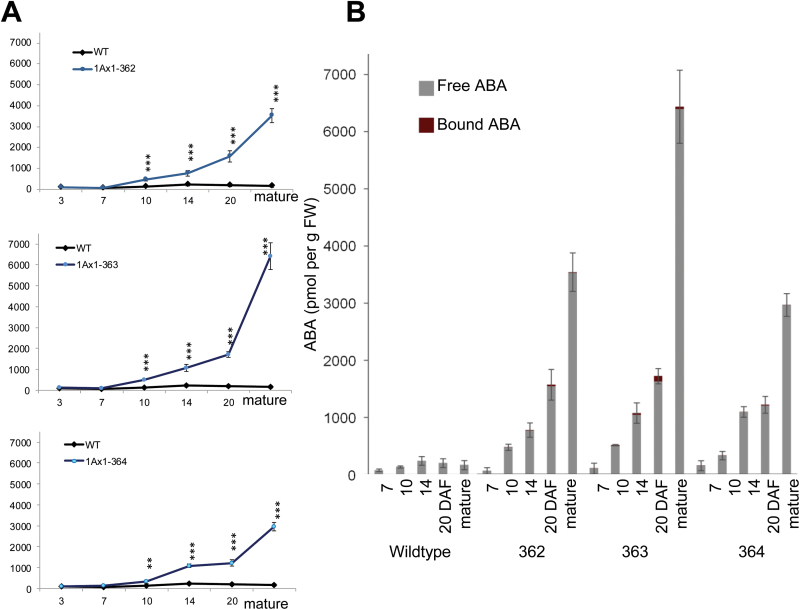
ABA content in developing grains. (A) Means ±SD of ABA-amounts in immunomodulated caryopses of aABA-lines 362, 363 and 364 during development, *n*=5. (B) Amounts of free ABA and ABA complexed with anti-ABA scFv in developing caryopses of immunomodulated lines 362, 363 and 364. Data are means ±SD and were calculated after Neri *et al.* (1996). Significant differences after ANOVA, **, *t*<0.01; ***, *t*<0.005. (This figure is available in colour at *JXB* online.)

Levels of ABA in mature immunomodulated grains increased dramatically as much as 10- to 40-fold, with line 363 showing the largest increase (Fig. 2D). For all three lines, ABA levels in developing grains were not different from the wild type at 3 and 7 DAF but were increasingly higher from 10 DAF until maturation (Fig. 3A). Such high increases of ABA levels have previously been observed in tobacco leaves (Wigger *et al.*, 2002) and seeds (Phillips *et al.* 1997) and in pea seeds (Radchuk *et al.* 2010a) expressing an anti-ABA antibody. The dramatic increase of ABA could be due to (i) increased rate of synthesis, (ii) decreased catabolism or (iii) reduced conjugation within the seeds. Since the anti-ABA antibody sequesters ABA in the ER compartment, it is conceivable that a feedback mechanism regulating ABA synthesis may be activated. However, it can also not be excluded that ABA is masked by the antibody, which prevents catabolism.

Amounts of free versus bound ABA in immunomodulated grains were calculated using concentrations of the scFv:ABA complex, total ABA concentrations, anti-ABA-scFv concentration and the dissociation constant Kd (Phillips *et al.* 1997; Radchuk *et al.* 2010a), (Fig. 3B). Accordingly, almost all ABA was present in the free unbound form. This is due to the fact that the molarity of the ABA in the caryopses is ~300-fold higher compared to the anti-ABA scFv, while binding of antibody and ABA occurs at nearly equimolar concentration to that of the antibody. Therefore, on average at 20 DAF, 99.4% of the antibody but only 5.6% of the ABA is bound (Fig. 3B).

### Phenotypical characterization of anti-ABA barley plants

**Fig. 4.**
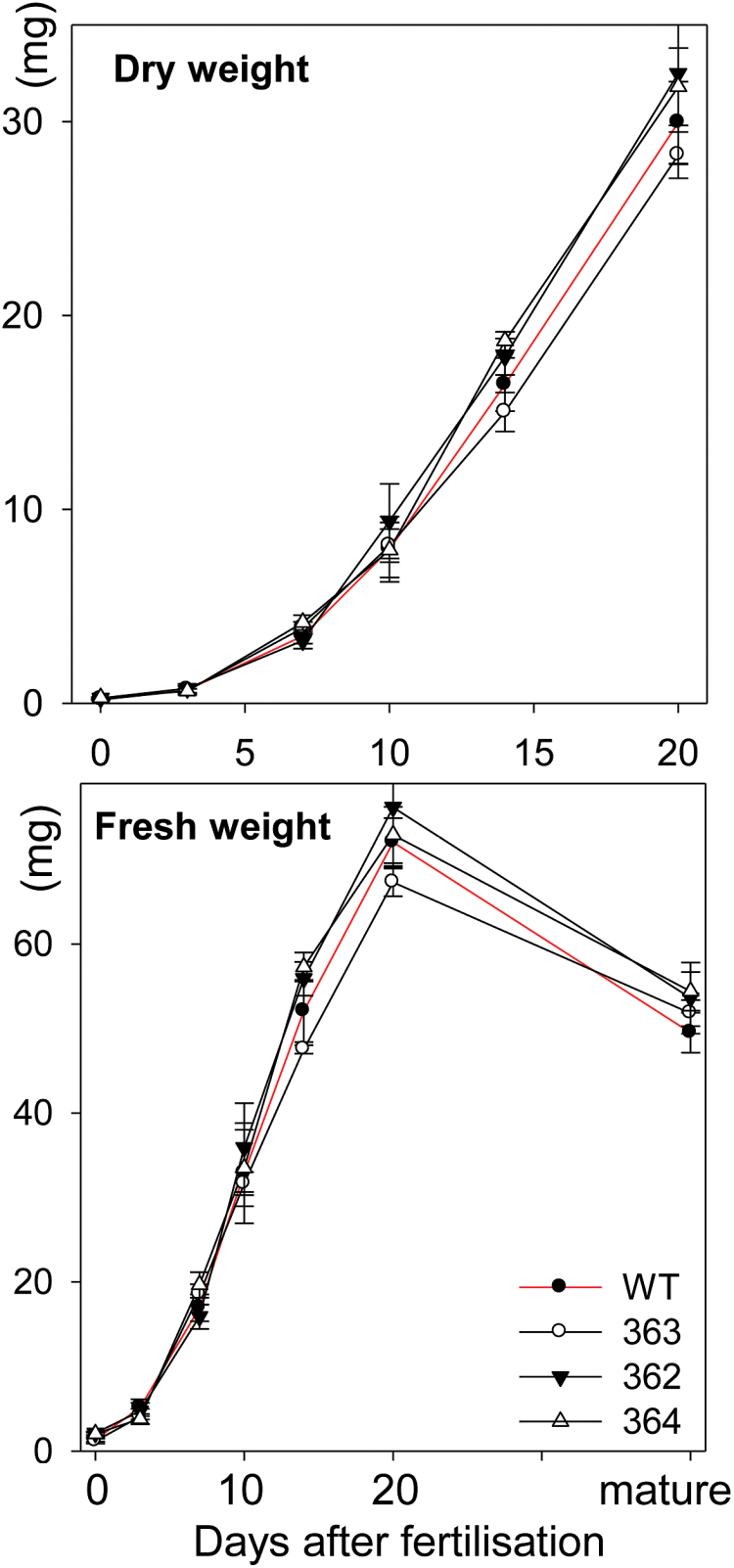
Growth curves of transgenic grains. Fresh weight and dry weight accumulation of developing caryopses of lines 362, 363, 364 and the wild type, means ±SD, *n*=40. (This figure is available in colour at *JXB* online.)

None of the three lines expressing the 1Ax1-promoter-antiABA construct showed any macroscopic, vegetative phenotype different from wild-type Igri after cultivation in greenhouses. Fresh weight and dry weight accumulation of caryopses were not significantly different between the anti-ABA lines and the wild type (Fig. 4) although there was a trend towards lower values for line 363, showing the greatest accumulation of scFv and ABA.

Surprisingly, a significant portion of the ABA-immunomo dulated grains showed a kink-like phenotype, from ~10 DAF onwards. These grains seemed to be impressed at the dorsal side and sections revealed that this is due to missing or dissolved endosperm cells (Supplementary Fig. S2A–H). The occurrence of this phenotype was observed in three independently grown plant sets and its frequency was from 2% to 10%, with line 363 showing the highest value (Supplementary Fig. S2I).

**Fig. 5.**
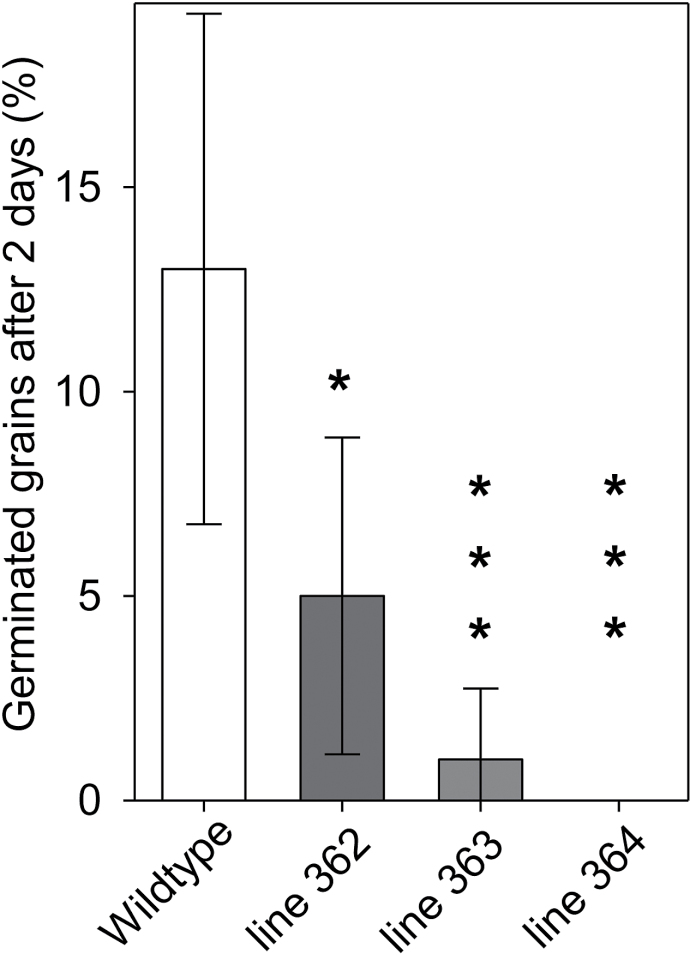
Germination assay. Percentage of germination of immunomodulated and wild-type grains, 2 d after imbibition, ±SD. A standardized germination test was performed starting with eight replicates of 25 grains per line. Significant differences after ANOVA, *, *t*<0.05; ***, *t*<0.005; *n*=25, *n*=8.

ABA is well known to inhibit seed germination. Therefore, we tested whether the increased ABA contents in mature grains affects germination. Some 200 grains per line were imbibed and grains that did not germinate after 12 d were regarded as dormant. The proportion of dormant grains in the anti-ABA lines and the wild type was not significantly different. However, the number of grains that had germinated after 2 d was significantly lower for all the three immunomodulated lines compared to the wild type (Fig. 5). This indicates that germination is delayed in the ABA-immunomodulated grains.

Thousand grain weight of mature seeds, determined in two consecutive years in the greenhouse, was not different between immunomodulated and wild-type plants.

### Composition and metabolite contents of anti-ABA grains

Mature grain composition was analysed for all three lines and revealed no significant differences for starch concentration, percentages of total carbon and nitrogen and percentages of prolamins and globulins/albumins. Starch concentration was analysed in grains of line 363 during development and revealed slightly but significantly lower levels in caryopses at only 10 DAF (Supplementary Fig. S3).

**Fig. 6.**
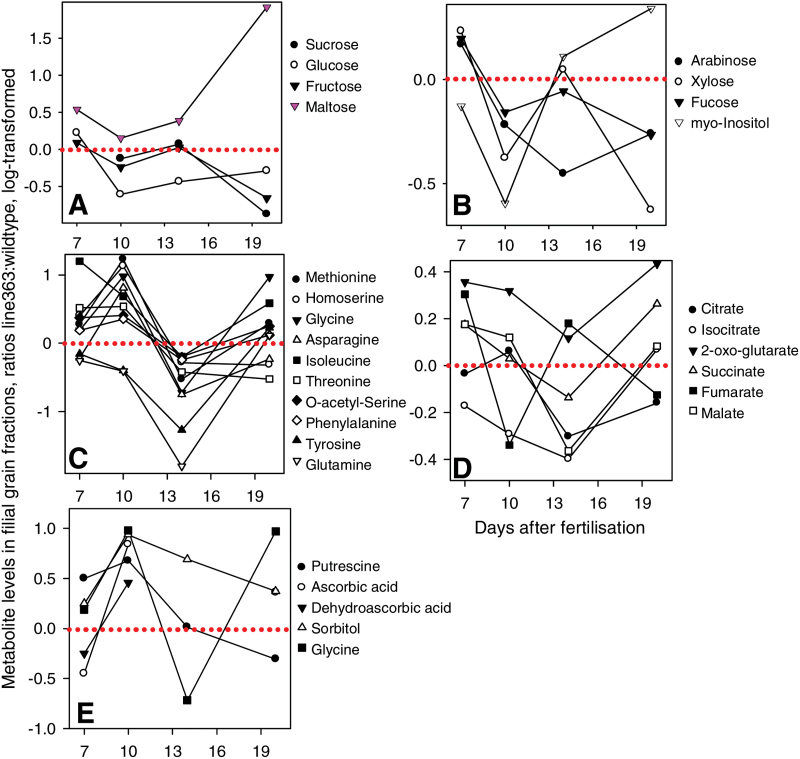
Metabolite profiling. Relative changes in metabolite levels in filial grain fractions of line 363 and wild type at 7, 10, 14 and 20 DAF measured by GC-MS. Data are presented as means (*n*=6) of log-transformed ratios, significant changes (*P*<0.05) of at least at one stage are given in bold face (see Supplementary Fig. S3), (A) soluble sugars, (B) cell wall sugars, (C) amino acids, (D) organic acids, (E) stress-related compounds. (This figure is available in colour at *JXB* online.)

Metabolite levels were measured in the filial fractions of immunomodulated grains of line 363 at 7, 10, 14 and 20 DAF using metabolite profiling of a fraction enriched for primary metabolites by gas chromatography-mass spectroscopy (GC-MS), (Erban *et al.*, 2007). In total, 100 assigned metabolites could be determined (Supplementary Table S1). Whereas the levels of the free sugars sucrose, glucose and fructose were clearly lowered compared to the wild type, those of maltose were increased, especially at 20 DAF (Fig. 6A, Supplementary Fig. S3). Levels of sugars involved in cell wall biosynthesis, arabinose, xylose, fucose and myo-inositol were commonly lower in anti-ABA grains, primarily at 10 DAF (Fig. 6B, Supplementary Fig. S3). Free sugars were also measured in whole caryopses of lines 362, 363 and 364 at 14 DAF by an enzymatic assay. All three aABA lines display decreased hexose levels during early maturation (14 DAF) and also increased sucrose to hexose ratios (Supplementary Fig. S4A). Since hexose levels decrease and sucrose to hexose ratios increase during pre-storage to storage phase (Weschke *et al.*, 2000), this indicates an advanced developmental stage compared to the wild type.

Amounts of free amino acids were somewhat disparate between anti-ABA and wild-type grains. However, a larger set of ten amino acids, among them the most abundant forms Asn and Gln, revealed commonly higher values at 10 DAF and lower levels at 14 DAF (Fig. 6C). Free amino acids in the filial fraction of caryopses of all three lines have been measured by ultra performance liquid chromatography (UPLC). The profiles of the major free amino acids during development compared to the wild type were very similar between the three lines (Supplementary Fig. S4B). Moreover the UPLC-generated values fit quite well to these generated by GC-MS for line 363 (Fig. 6C).

Levels of glycolytic intermediates between anti-ABA and wild-type grains were inconsistent, with either unchanged levels of hexose-phosphates but lower amounts of phosphoenolpyruvate (14 DAF) and higher levels of 3-phospho-glycerate (Supplementary Fig. S3). Whereas amounts of citrate and iso-citrate were lower in anti-ABA grains at 14 DAF, that of 2-oxo-glutarate were higher at 10 and 20 DAF (Fig. 6D, Supplementary Fig. S3). Noticeably, amounts of putrescine, ascorbate, sorbitol and glycine, which are related to stress response and tolerance (Kempa *et al.*, 2008; Maruyama *et al.*, 2014), exhibited higher levels in anti-ABA grains at certain stages (Fig. 6E). Also the amounts of stress-inducible gamma-aminobutyric acid were elevated in the filial caryopses of all three lines (Supplementary Fig. S5).

### Transcript profiling of developing anti-ABA filial grain fractions

**Fig. 7.**
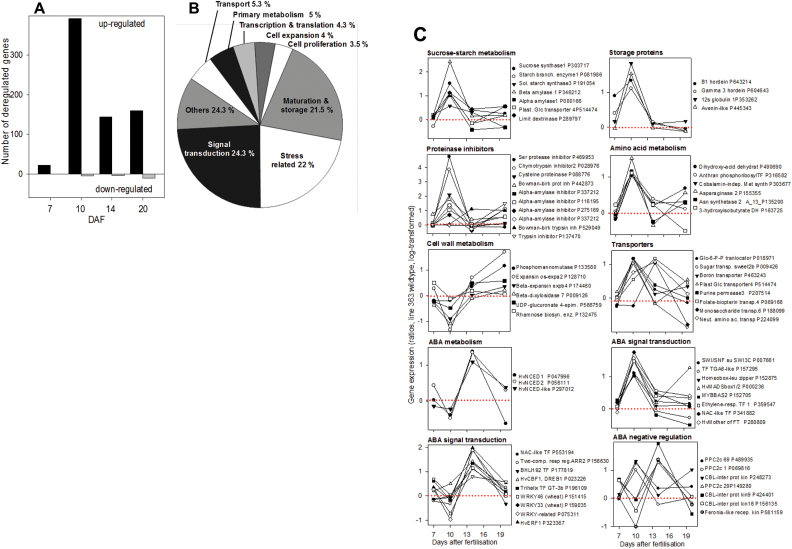
The effect of ABA immunomodulation on gene expression in filial fraction of caryopses of line 363. (A) Numbers of genes up- or down-regulated in filial fractions of caryopses of line 363 at 7, 10, 14, and 20 days after fertilisation (DAF) (B) Categorization of up-regulated genes in the filial grain fraction of line 363 compared to the wildtype IGRI (C) Expression pattern of genes related to specific pathways and physiological states in filial grain, given as ratios. Line 363 to wild type, filial fractions and log-transformed data are extracted from Supplementary Table S2. Sequence IDs (see Supplementary Table S2) are given as P[xxxxx]. (This figure is available in colour at *JXB* online.)

To analyse global changes of gene expression due to ABA immunomodulation, comparative transcript profiling was performed for filial grain fractions of line 363 and wild type at 7, 10, 14 and 20 DAF using the 4×44K Barley Gene Microarray (www.agilent.com). Genes were selected whose expression was significantly up- or down-regulated by a factor of at least two and in at least one stage (*P*<0.05, three biological replicates). Since transcript abundances do not necessarily reflect transcriptional activity, protein content or enzyme activity, all statements on gene identity and function have to be considered as ‘putative’. For reasons of simplicity, higher or lower transcript levels were subsequently referred to as down- or up-regulated. In total, 682 genes were de-regulated in anti-ABA grains (Supplementary Table S2). The distribution across the developmental stages revealed that the number of up-regulated genes is very low at 7 DAF but increased by 18-fold from 7 to 10 DAF and decreased again by 2-fold at 14 and 20 DAF (Fig. 7A). Therefore, ABA immunomodulation obviously generates a general up-regulation of gene expression for >97% of all de-regulated genes, especially at 10 DAF. The categorization of up-regulated genes revealed three major categories: signal transduction (24.3%), stress response and tolerance (22%) and maturation and storage (21.5%). Other up-regulated categories accounted for only 3.5–5.3%, such as genes related to transport, primary metabolism, cell proliferation and expansion, transcription and translation (Fig. 7B).

### Deregulation of storage-associated gene expression in anti-ABA grains

The transition stage of barley grain development is characterized by increased metabolic activity related to starch and storage protein synthesis while growth events such as cell proliferation decreased. At 10 DAF, immunomodulated grains displayed transient transcriptional up-regulation of enzymes of sucrose to starch metabolism such as sucrose synthase, starch synthases and limit dextrinase. In parallel, β-amylase-1, α-amylase-1 and a plastidic glucose transporter were also more highly expressed in anti-ABA grains at 10 DAF. This suggests considerable starch turnover in the anti-ABA grains. However, at later stages, levels of these transcripts remained unchanged (Fig. 7C).

Similar to genes associated with starch metabolism, storage protein genes, such as hordeins, globulins and avenins were also transiently up-regulated at 10 DAF together with a set of maturation-associated protease-inhibitor genes such as α-amylase- and trypsin-inhibitors. The majority of enzymes of amino acid metabolism was also up-regulated at 10 DAF, among them dihydroxy-acid dehydratase, anthranilate phosphoribosyl-transferase, methionine synthase and asparagine synthetase-2. The results suggest a general stimulation of storage-related gene transcription in the ABA-immunomodulated caryopses, however only transiently at 10 DAF. At the same time, at 10 DAF, a set of six enzymes involved in cell expansion and the biosynthesis of cell wall-related sugars was transiently down-regulated in anti-ABA grains, among them two expansins, phospho-mannomutase, UDP-glucuronate-4-epimerase and rhamnose-biosynthesis enzyme. Several genes related to sugar and metabolite transport were up-regulated at either 10 DAF, 14 DAF (Fig. 7C) or at 20 DAF (Supplementary Table S2).

### Deregulated gene expression related to hormone functions and signal transduction

ABA-immunomodulated caryopses accumulate high amounts of ABA (Fig. 3). Three genes, upregulated at 14 DAF in caryopses of line 363, encode 9 cis-epoxy-carotenoid-dioxygenases, HvNCED1, HvNCED2 and HvNCED-like (Fig. 7C). NCEDs were shown to be rate-limiting for ABA biosynthesis (Thompson *et al.*, 2000; Qin and Zeevaart, 2002). Obviously, the up-regulated HvNCEDs were involved in the over-shooting biosynthesis of ABA observed in immunomodulated caryopses.

ABA integrates grain development with stress response and adaption and functions through various positive and negative signalling elements. In anti-ABA grains eight related genes are upregulated at 10 DAF (Fig. 7C). The SWI3 subunit of the SWI/SNF chromatin-remodelling complex might be connected with ABA signalling pathways, suggesting that chromatin-remodelling is involved in ABA response to abiotic stress (Saez *et al.*, 2008). The TGA6-like transcription factor could be involved in pathogenesis-related gene expression and pathogen resistance as seen by knock-out analysis in Arabidopsis (Zhang *et al.*, 2003). The homeobox-leucine zipper protein might be involved in regulating development in response to environmental conditions. In sunflower, such a member, Hahb-4, is rapidly and strongly induced by drought and ABA (Gago *et al.*, 2002). The HvMADS box1/2 (HvOS1/2) protein is related to type I MADS-box genes. HvOS1/2, exhibiting differential expression during barley seed development, is induced by ABA and the protein is detected in specific endosperm subcompartments. DNA methylation differences associated with gene expression suggests epigenetic regulation according to Arabidopsis type I MADS box protein (Kapazoglou *et al.*, 2012). The Myb-BAS2 protein potentially functions as transcriptional activator in ABA-inducible gene expression under drought stress (Abe *et al.*, 2003). The ethylene-response factor-1-protein potentially regulates gene expression under abiotic/biotic stress. In wheat, TaERF1 is induced by drought, salinity, low-temperature, ABA, ethylene and salicylic acid. When overexpressed, stress-related genes are activated leading to improved pathogen and abiotic stress tolerance (Xu *et al.*, 2007). The NAC-like transcription factor might be involved in plant development and stress responses. In rice, ONAC045 was induced by drought, high salt, and low temperature stresses. Arabidopsis overexpressing ONAC045 showed enhanced tolerance to drought and salt (Zheng *et al.*, 2009). The HvMFT, MOTHER OF FT AND TFL1 (MFT) belongs to phosphatidylethanolamine-binding proteins and is known as key regulators of flowering. In wheat and soybean, an MFT homologue negatively regulates germination and is transcriptionally induced and repressed by ABA and gibberellic acid, respectively (Nakamura *et al.*, 2011; Li *et al.*, 2014).

Another set of ten genes, potentially involved in ABA signalling, is up-regulated at 14 DAF. Three are WRKY-like transcription factors, which are known as key components of ABA signalling. Its target genes are involved in ABA-effects and drought stress response (Rushton *et al.*, 2012). A homologue of the bHLH92 transcription factor, in Arabidopsis is strongly salt- and drought-inducible and dependent on ABA (Jiang *et al.*, 2009). A two-component-response regulator, ARR2, could act as transcriptional activator and is up-regulated at 14 DAF. In Arabidopsis, subunits of two-component signalling system are involved in cold stress response (Jeon *et al.*, 2010). Transcriptionally stimulated HvCBF1, which is a member of CBF/DREB1 family genes, could be involved in signalling low temperature, drought and salinity tolerance in plants including barley (Wu *et al.*, 2011). The trihelix-GT3b factor belongs to founding members of the trihelix transcription factor family. Expression of some of these genes is induced by ABA, salt, drought and cold, and overexpression in Arabidopsis increases tolerance to salt, drought and freezing (Kaplan-Levy *et al.*, 2012).

Seven genes, which encode potential negative regulators of ABA signal transduction, are up-regulated at 10 and 14 DAF in anti-ABA grains. Three encode protein phosphatases 2c (PP2C), which interact with kinases, phosphatases, transcription factors and metabolic enzymes thereby inhibiting ABA signals (Yoshida *et al.*, 2015). Three other candidates are related to CBL-interacting protein kinases (CIPKs). This signalling pathway is Ca^++^-related and responds strongly to abiotic and biotic environmental stimuli. Several CIPK-members have been described as negative regulators of ABA functions (Kim *et al.*, 2003; Pandey *et al.*, 2004). Finally, the FERONIA-like receptor kinase, which was described as positive regulator of auxin-promoted growth, could repress ABA response through activation of ABI2, a PP2C member suppressing ABA responses (Yu *et al.*, 2012).

## Discussion

Abscisic acid generally accumulates in response to different biotic and abiotic stresses, delays growth and development and induces stress-tolerance-related gene expression. In seeds, ABA accumulates during the transition to the filling phase and triggers seed maturation, storage activity and the biosynthesis of stress signalling- and tolerance-related proteins (Finkelstein *et al.*, 2002). During late maturation and desiccation, ABA promotes the synthesis of late embryogenesis abundant (LEA) proteins, prevents precocious germination and confers desiccation tolerance and dormancy. ABA functions via a balanced network of biosynthesis and degradation, interaction with other hormones and by integration within a system of negative and positive transcriptional regulators (Raghavendra *et al.*, 2010). Immunomodulation is a suitable tool to alter the hormonal status in seeds, without directly intervening with biosynthesis, degradation or signalling pathways (Conrad and Manteuffel, 2001). In such a way, an anti-ABA antibody gene was directed into barley, whereupon the 1Ax1 promoter mediates strong expression in the endosperm while the anti-ABA antibody is retained within the ER and sequesters ABA.

### Anti-ABA caryopses accumulate an excess of free ABA

The caryopses of transgenic plants respond to anti-ABA antibody gene expression with increased production of ABA, evidently, by up-regulation of different NCEDs (Fig. 7C). While there is no indication for activated ABA degradation or inactivation, the exact localization of this surplus ABA is still unclear. One possibility is vacuolar sequestration, since transcriptional up-regulation of vacuolar ATPase and H^+^-PPiase (Supplementary Table S2), which are known targets for ABA regulation (Fukuda and Tanaka, 2006), improves energized vacuolar transport.

The calculation of free versus antibody-bound ABA reveals a large excess of the free hormone, whose levels increase greatly in the caryopses from 10 DAF onwards. Thus, the situation in anti-ABA barley clearly differs from those of other models, where immunomodulation has been used to alter the ABA status. Whereas in Arabidopsis (Phillips *et al.* 1997), tobacco (Artsaenko *et al.*, 1995) and pea (Radchuk *et al.* 2010a), anti-ABA antibody gene expression decreased free ABA, its levels are increased in the anti-ABA caryopses described in the current study. This is due to the fact that aABA grains produce high ABA levels from which only a small fraction is bound by the antibody. However, since gene expression and protein accumulation of the anti-ABA antibody starts at 7 DAF (Fig. 2B) when endogenous ABA is still very low (Weier *et al.* 2014), transient ABA limitation at that early stage cannot be excluded, which might induce ABA over-production.

### Anti-ABA caryopses reveal broad stimulation of storage and maturation-related activities

At the stage when free ABA starts to accumulate (10 DAF), early cell divisions in the caryopses have already terminated and grains are in the transition stage, which is characterized by transcriptional and metabolic phase changes (Kohl *et al.*, 2015). ABA is obviously involved in this switch and decreasing its levels by immunomodulation during this critical period attenuates phase transitions, delays maturation and seed development in tobacco (Phillips *et al.* 1997), bean (Radchuk *et al.*, 2006) and pea (Radchuk *et al.* 2010a). Metabolite and transcript profiling in anti-ABA grains reveal specific changes, which conform to triggered and enhanced ABA functions. This is evident on different levels, such as transcriptional up-regulation of sucrose-to-starch metabolism, storage protein synthesis and ABA-related signal transduction, and leads to gene-induction associated to grain maturation, including proteinase inhibitors, LEA- and numerous stress-associated genes. (Fig. 7C, Supplementary Table S2). ABA is a well known activator of sucrose to starch conversion in rice (Wang *et al.*, 2015) and barley (Sreenivasulu *et al.*, 2006). LEA and proteinase inhibitor genes are responsive to ABA and stress (Skriver and Mundy, 1990). Several up-regulated genes, which are targets for ABA regulation, are involved in the energization of transport, including V-ATPase and H^+^-PPiase, (Fukuda and Tanaka, 2006) and in mitochondrial energy production such as the mitochondrial ADP:ATP carrier, ATP-synthase (Supplementary Table S2). In Arabidopsis, V-ATPase is required for efficient nutrient acquisition (Krebs *et al.*, 2010). Obviously, ABA promotes nutrient transport during grain maturation via stimulation of membrane energization.

ABA-stimulated storage activity at 10 and 14 DAF is also reflected in metabolite levels. The decrease in free sugars, especially that of glucose, may indicate enhanced demand for starch biosynthesis. Lower levels of citrate and iso-citrate and higher levels of 2-oxo-glutarate could reflect an increased usage of organic acid, particularly for transamination reactions. Levels of many free amino acids are increased at 10 DAF but decreased at 14 DAF (Fig. 7C, Supplementary Fig. S5). This could point to increased biosynthesis at 10 DAF, generated by increased ABA. Subsequently, the lower levels at 14 DAF could indicate limitations due to stimulated storage protein synthesis. In various plant species, ABA and/or drought stress effect accumulation of free amino acids (Urano *et al.*, 2009; Bowne *et al.*, 2012). In accordance, enzymes of amino acid metabolism are up-regulated in anti-ABA caryopses at 10 DAF (Fig. 7C, Supplementary Table S2), such as dihydroxyacid dehydratase involved in the biosynthesis of branched-chain amino acids, whose levels increased under drought stress in wheat (Bowne *et al.* 2012). Anthranilate phosphoribosyl-transferase, asparagine synthetase and methionine synthase are responsive to wounding, salt and cold/drought/salt/ABA, respectively (Pratelli and Pilot, 2014). Grains of three aABA lines display decreased hexose levels and increased sucrose to hexose ratios during early maturation at 14 DAF (Supplementary Fig. S3). High amounts of free hexoses in barley as well as in other seeds indicate a young stage of development, whereas the ratio of sucrose to hexose increases during transition to maturation (Weschke *et al.*, 2000; Weber *et al.*, 2005). The fact that sucrose to hexose ratios are significantly increased in the aABA caryopses at 14 DAF indicate advanced developmental and precocious maturation compared to the wildtype.

### ABA accumulation induces precocious maturation integrated with stress response

The data show that in anti-ABA caryopses levels of free ABA are increased from 10 DAF, which leads to a broad stimulation of storage and maturation-related gene expression.

Unexpectedly, along with starch biosynthesis, several α- and β-amylases are also up-regulated in anti-ABA caryopses, accompanied with lower starch contents at 10 DAF (Supplementary Fig. S3). This could be due to enhanced stress signalling. Similarly, in rice, starch degradation is transcriptionally up-regulated in response to cold or dehydration (Maruyama *et al.* 2014) pointing to an interplay of ABA and stress signalling. Anti-ABA grains display broad constitutive gene induction related to different biotic and abiotic stresses mainly at 10 and 14 DAF (Fig. 7C, Supplementary Table S2). Most of the related genes are ABA- and/or stress-inducible (Ingram and Bartels, 1996) and include alcohol and aldehyde dehydrogenases, peroxidases, chaperones and glutathione-S-transferase, involved in chemical detoxification. The fact that anti-ABA caryopses do not suffer from stress implicates that the increased ABA mimics such an effect and causes gene expression related to stress response and tolerance. It further shows that in anti-ABA grains the precocious accumulation of ABA generates an integrated response of stress and maturation.

In parallel with untimely induction of storage activities on the levels of transcripts and metabolites, cell wall metabolism is down-regulated, which is reflected by lower levels of cell wall sugars (Supplementary Fig. S3) at 10 DAF and transcriptional down-regulation of expansins and several enzymes related to cell wall metabolism (Fig. 7C). Such negative effects on cell wall metabolism could be explained by the general growth-inhibiting effect of ABA. Similarly, in squash hypocotyls ABA inhibits the synthesis of cell wall polysaccharides (Wakabayashi *et al.*, 1991). Also in maize leaves, ABA suppresses leaf elongation rate (Cramer and Quarrie, 2002). These deficiencies in cell wall biosynthesis can also explain the observed kink-like phenotype of a considerable fraction of anti-ABA grains, which may be derived from failure to synthesize intact cell walls. It is tempting to speculate that in the anti-ABA caryopses the advanced increase of ABA during the transition phase negatively interferes with cell expansion and development and induces precocious and untimely maturation.

### ABA sensing and signalling in anti-ABA grains integrates positive and negative regulation

Despite the stimulating effect of elevated ABA on storage activities and grain maturation, grain weight and composition of mature grains are unchanged in anti-ABA plants, although germination is somewhat delayed. This implicates that the advanced maturation triggered by increased ABA is partially balanced at later development, so that maturating grains are not largely affected. In this respect it is noticeable that the stimulation of respective gene expression occurs only transiently at 10 and 14 DAF, whereas at later stages (20 DAF) there are just minor differences (Fig. 7C). It is known that ABA induces specific mechanisms to desensitize its signalling efficiency (Phillips *et al.*, 2007), which will result in decreased perception of ABA levels (Reyes and Chua, 2007). Transcript profiling in the anti-ABA caryopses reveals the activation of a network of positive and negative regulators involved in ABA-related signalling (Fig. 7C, Supplementary Table S2). Several of these elements are possibly involved in triggering transcriptional changes, which are required to switch development during the transition stage towards activating maturation. Such factors include subunits of the SWI/SNF chromatin remodelling complex, homeobox-leucine-zipper proteins and HvOS1/2, implicating that ABA can alter development on the levels of chromatin and DNA methylation and/or epigenetics (Saez *et al.* 2008; Kapazoglu *et al.* 2012). A broad range of elements, up-regulated in the anti-ABA grains, involves stress signalling such as TAG6, Myb BAS2, bHLH92, HvCBF1, Trihelix GT3b, HvERF1, NAC- and WRKY-like transcription factors (Fig. 7C). However, at least seven up-regulated signalling factors in anti-ABA grains potentially might antagonize ABA functions, including PP2C, CBL-CIPK and the FERONIA-like receptor (Pandey *et al.* 2004; Yu *et al.* 2012; Yoshida *et al.* 2015) Also WRKY-like transcription factors can repress ABA signalling (Zhang *et al.*, 2015).

In summary, there is evidence that especially during the later development of grain filling the repression of ABA-signalling could antagonize the effect caused by its over-shooting production. This ultimately leads to normalization of grain development and grain filling and generates mature grains with nearly unchanged dry weight and composition. Such compensation highlights the enormous physiological and metabolic flexibility of barley grain development to adjust the effects of high ABA levels in the anti-ABA grains in order to ensure and maintain proper grain development.

## Supplementary data

Supplementary data are available at *JXB* online.

Fig. S1. Southern analysis of independent aABA lines.

Fig. S2. Presentation of the kink-like phenotype in ABA-immunomodulated grains by sectioning and frequency of the phenotype in ABA-immunomodulated grains.

Fig. S3. Metabolite profiling. Relative changes in metabolite levels in filial grain fractions of line 363 and wild type at 7, 10, 14 and 20 DAF measured by GC-MS.

Fig. S4. Relative concentrations of hexoses (sum of glucose and fructose), sucrose and sucrose to hexose ratios in the aABA grains of lines 362, 363 and 364.

Fig. S5. Free amino acids in aABA grains of lines 362, 363 and 364 measured by UPLC.

Table S1. Analysis of differentially accumulated metabolites measured by GC-MS based on profiling of the filial grain fractions of ABA-immunomodulated compared to wild type grains at 7, 10, 14 and 20 DAF.

Table S2. List of differentially expressed genes in the filial fraction of grains of line 363.

Supplementary Data
