# Supplementary material for: Increasing abscisic acid levels by immunomodulation in barley grains induces precocious maturation without changing grain composition
Source: J Exp Bot. 2016 Mar 7;67(9):2675–87. doi: 10.1093/jxb/erw102 (PMC4861016; doi:10.1093/jxb/erw102)
Supplement: Supplementary Data [file supp_67_9_2675__index.html]

Increasing abscisic acid levels by immunomodulation in barley grains induces precocious maturation without changing grain composition — Increasing abscisic acid levels by immunomodulation in barley grains induces precocious maturation without changing grain composition — Supplementary Data 

# Increasing abscisic acid levels by immunomodulation in barley grains induces precocious maturation without changing grain composition

## Supplementary Data

Data files

- supplementary\_figures\_S1\_S5.pdf - Supplementary Data
- supplementary\_table\_S1.xlsx - Supplementary Data
- supplementary\_table\_S2.xlsx - Supplementary Data
