# Supplementary material for: Increasing abscisic acid levels by immunomodulation in barley grains induces precocious maturation without changing grain composition
Source: J Exp Bot. 2016 Mar 7;67(9):2675–87. doi: 10.1093/jxb/erw102 (PMC4861016; doi:10.1093/jxb/erw102)
Supplement: Supplementary Data [file supp_erw102_supplementary_figures_S1_S5.pdf]

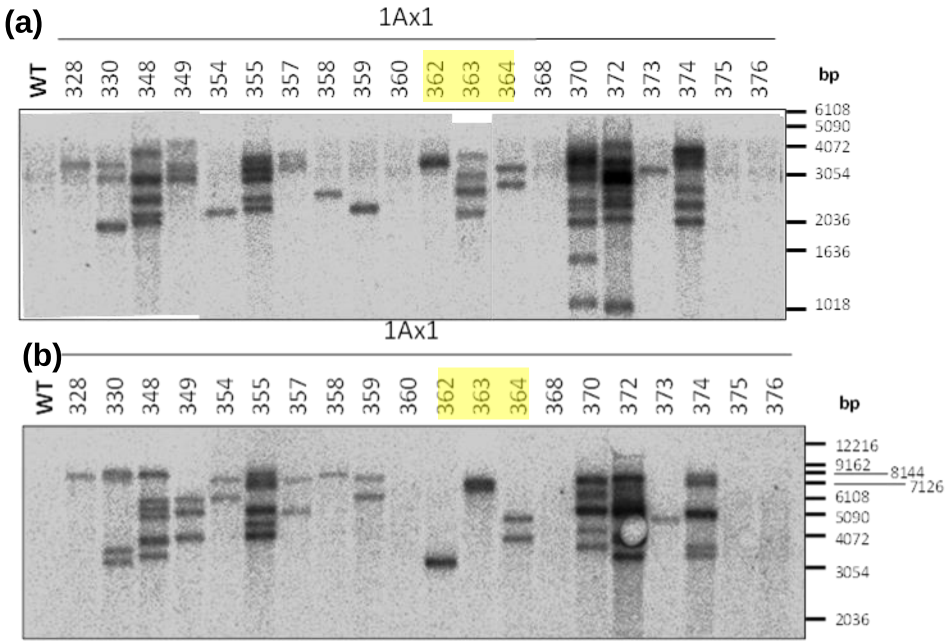

Figure S1, Southern blot analysis of anti-ABA plants.
10 µg of genomic DNA from leaves were cut by *Bam*HI (a) and *Hin*dIII (b) separated by elctrophoresis and immobilised on nylon filters. Hybridisation was done under stringent conditions using a 447 basepair (bp) fragment of the anti-ABA scFv-Gene labelled with ^{32}P. Yellow-shaded lines were selected for analysis.

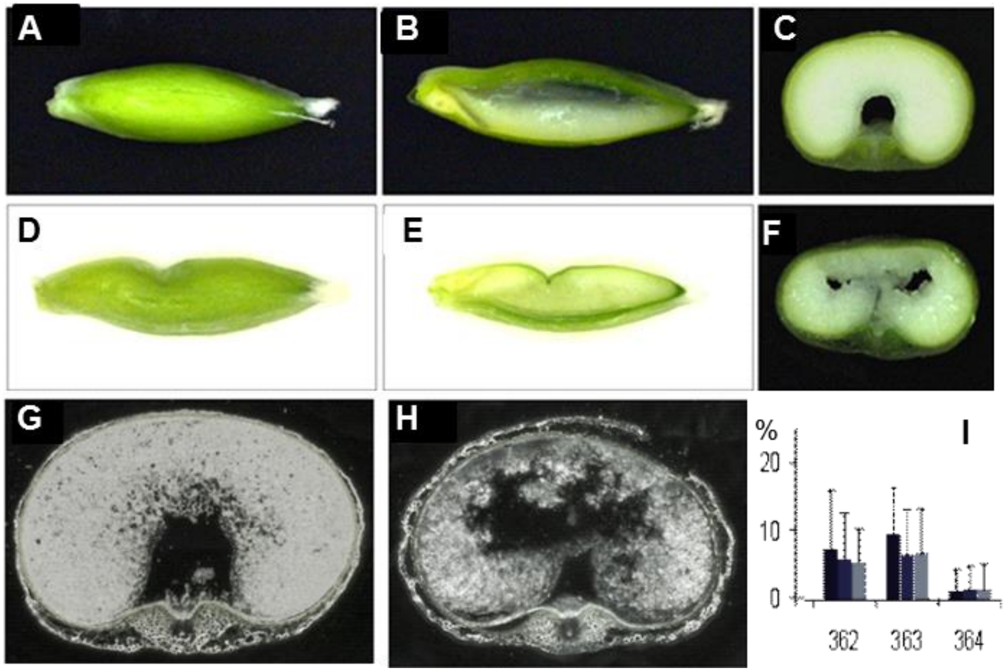

Figure S2, Demonstration of the kink-like phenotype.
A-C, wildtype, **D-F,** aABA grains, A, D, lateral view, B, E, longitudinal sections, c, f, cross-sections through the kink region, **G-H** 25µm cryo-sections through the kink region at 14 DAF of wildtype (G) and aABA-line (H), generated by Cryostat OTF, Bright Instruments, Cambridgeshire, GB, **I**, percentage of the kink-like phenotype in lines 362, 363, 364 (three independent growings), the kink-like phenotype was not observed in wildtype grains.

SUGARS / CARBOHYDRATES	**7**	**10**	**14**	**20 DAF**
Sucrose	0.42	-0.12	0.07	**-0.87**
Glucose	**0.22**	**-0.61**	**-0.44**	**-0.29**
Fructose	0.09	-0.24	0.03	**-0.65**
Maltose	0.54	0.15	0.38	**1.92**
Starch*	0.40	**-0.30**	-0.06	0.06
Glucose-6-phosphate	-0.08	0.15	-0.06	0.14
Fructose-6-phosphate	-0.03	0.15	-0.06	0.01
Fructose-1-phosphate	0.07	0.02	0.22	0.08
3-Phospho-glycerate	0.01	**0.61**	-0.19	0.32
Glycerol-3-phosphate	0.19	**0.35**	0.12	0.29
Phosphoenolpyruvate	-0.03	-0.61	**-0.60**	0.47
Shikimiate	0.12	0.02	**-0.35**	0.18
Arabinose	0.17	**-0.22**	**-0.45**	**-0.26**
Xylose	**0.23**	**-0.38**	0.05	**-0.63**
Fucose	0.20	-0.16	-0.06	-0.27
myo-Inositol	-0.13	**-0.60**	0.11	**0.34**
Raffinose	0.07	-0.57	0.74	0.84
Galactinol	0.07	-0.44	0.04	0.30
Sorbitol	0.25	**0.94**	0.69	**0.37**
Trehalose	**0.33**	-0.01	0.89	0.37
1-Kestose	0.04	-0.42	-0.33	**0.98**
Beta-D-Fructofuranose	-0.09	**-0.57**	-0.12	-0.38

ORGANIC ACIDS				
Citrate	-0.04	0.06	**-0.30**	-0.16
Isocitrate	-0.17	-0.29	**-0.40**	0.07
2.oxo-glutarate	0.36	**0.32**	0.12	**0.44**
Succinate	0.18	0.03	-0.14	0.26
Fumarate	0.30	-0.34	0.18	-0.13
Malate	0.18	0.12	-0.36	0.08

AMINO ACIDS	**7**	**10**	**14**	**20 DAF**
Methionine	0.28	**1.23**	-0.52	0.29
Homoserine	0.40	**1.13**	-0.28	-0.32
Glycine	0.19	**0.98**	-0.72	**0.97**
Asparagine	-0.21	0.81	**-0.75**	-0.23
Isoleucine	**1.27**	0.69	-0.20	0.59
Threonine	0.52	0.54	-0.42	-0.52
O-acetyl-Serine	0.37	0.41	-0.20	0.25
Phenylalanine	0.19	0.36	-0.24	0.12
Lysine	**0.99**	0.84	0.57	0.63
2-amino Adipic acid	**0.35**	0.35	0.38	**1.92**
Valine	**0.56**	0.34	0.00	0.08
Alanine	0.09	0.20	0.35	**0.62**
Leucine	**0.39**	0.18	-0.03	**0.32**
Gaba	**0.45**	0.13	**1.00**	**0.46**
Cytosine	0.14	0.10	0.01	0.08
Glutamic acid	0.05	0.08	0.20	0.59
Serine	0.03	0.07	0.07	0.60
Arginine	-0.43	-0.11	**-0.21**	0.11
Proline	0.13	-0.17	-0.16	**0.94**
N-acetyl-Serine	0.30	-0.24	0.28	0.00
Ornithine	0.54	-0.24	-0.24	-0.06
Beta-Alanine	-0.05	-0.35	0.18	0.29
Tyrosine	-0.16	-0.40	**-1.27**	0.23
Glutamine	-0.25	-0.41	**-2.02**	0.12
Aspartic acid	-0.16	-0.55	-0.07	-0.09

OTHERS				
Putrescine	0.50	**0.68**	0.01	-0.31
Spermidine	0.06	-0.45	-1.41	-0.61
1,4-diamino Propane	**0.62**	0.24	-0.12	-0.21
Ascorbic acid	-0.45	**0.84**		0.36
Dehydroascorbic acid	-0.25	0.46		**1.09**
Glycerophosphoglycerol	0.09	0.33	**0.48**	**1.98**
Glyceric acid	0.29	-0.30	**0.54**	**0.50**
Indole-3-acetic acid	0.06	-0.36	-0.09	**-0.80**

Figure S3, Metabolite profiling.
Relative changes in metabolite levels in filial grain fractions of line 363 and wildtype at 7, 10, 14 and 20 DAF measured by GC-MS. Data are presented as means (n = 6) of log-transformed ratios, significant changes ($P < 0.05$) of at least at one stage are given in bold face, *, analysed by enzymatic assay.

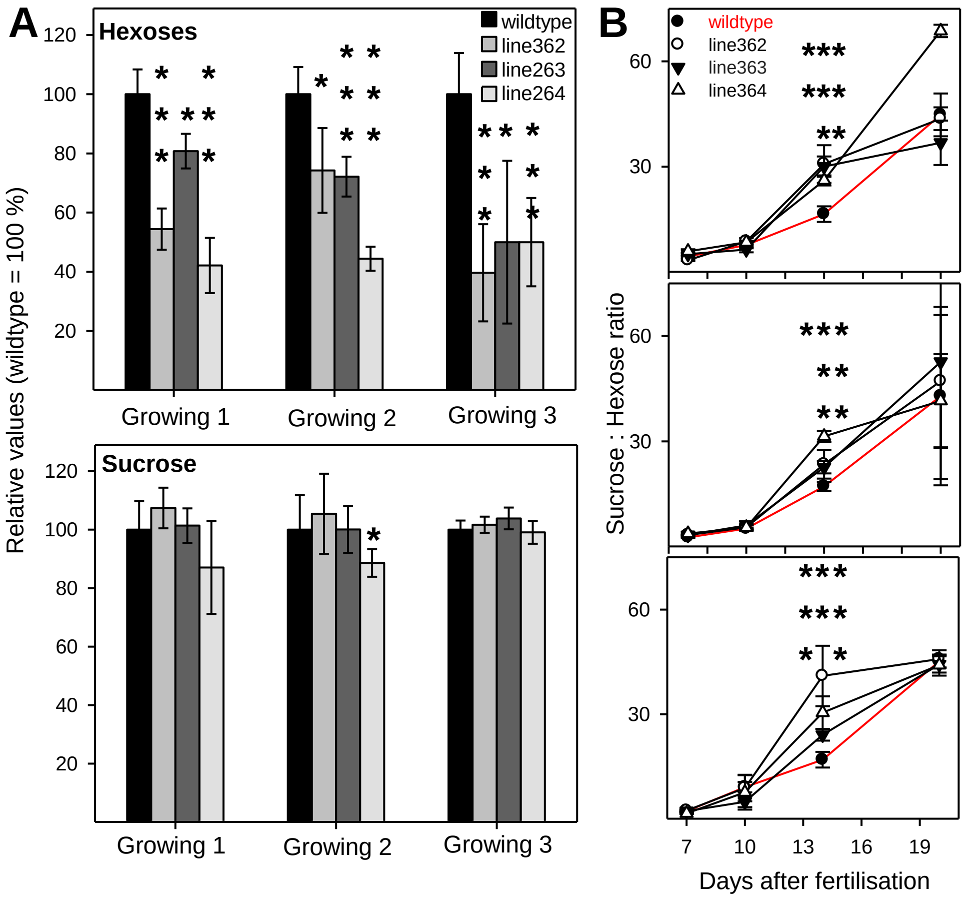

Figure S4, Free sugars in aABA-grains of lines 362, 363 and 364.

A, Relative concentrations of hexoses (sum of glucose and fructose) and sucrose in whole caryopses at 14 DAF of three independent growings ± standard deviation, measured by an enzymatic assay (Rolletschek *et al.,* 2002). Wildtype levels were set as 100 %. **B,** Sucrose to hexose ratios ± standard deviation in the aABA grains, calculated from data in (A). Statistical differences after single-factor ANOVA, * t<0,05; ** t<0,01; *** t<0,005.

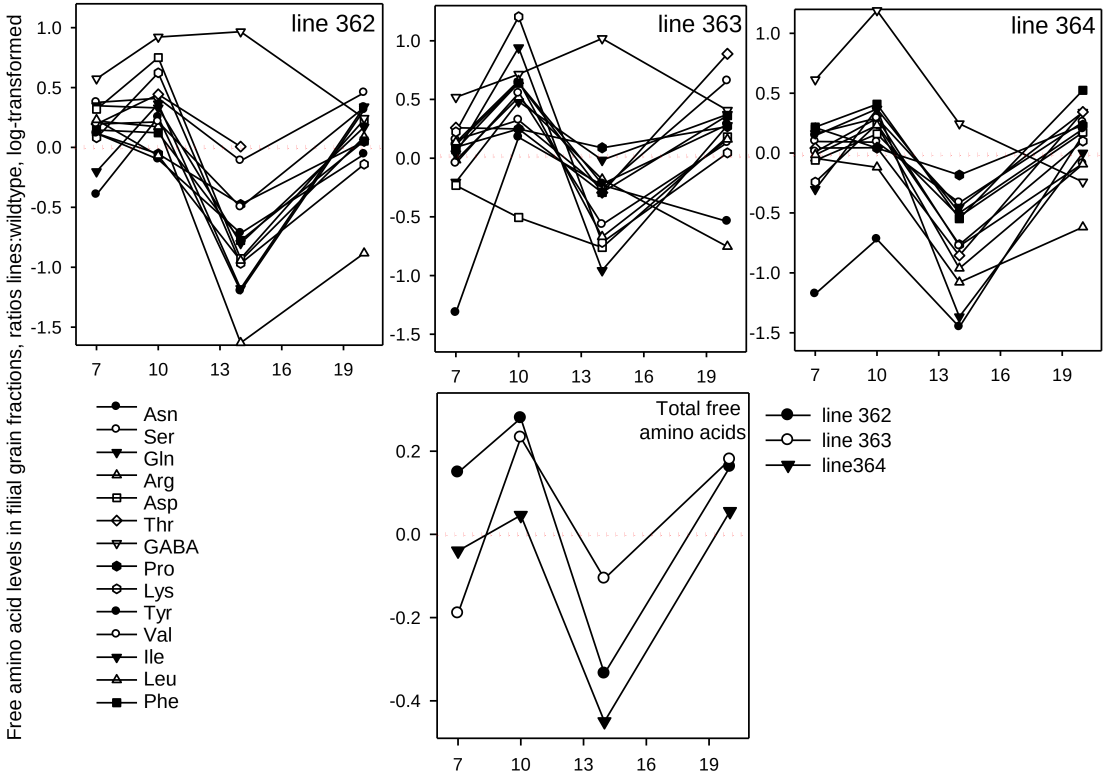

Figure S5, Free amino acids in aABA-grains of lines 362, 363 and 364.
Free amino acids were measured by Ultra Performance Liquid Chromatography (UPLC) from the filial fraction of aABA-grain of lines 362, 363 and 364 after a method described in Thiel et al., (2009).
